# Association between depressive status and mild cognitive impairment in middle-aged and elderly Chinese adults from CHARLS study

**DOI:** 10.3389/fpsyt.2025.1516341

**Published:** 2025-02-13

**Authors:** Caijuan Wei, Jinyu Zhao, Rui Hu, Xingli Wei

**Affiliations:** ^1^ Hospice Service Unit, The First Hospital of Lanzhou University, Lanzhou, China; ^2^ The First Clinical Medical School, Lanzhou University, Lanzhou, China; ^3^ Department of Nephrology, The First Hospital of Lanzhou University, Lanzhou, China

**Keywords:** CHARLS, mild cognitive impairment, depression, depressive status, restricted cubic spline

## Abstract

**Background:**

The potential association between depressive status and the risk of mild cognitive impairment (MCI) remains unclear, especially in the absence of prospective evidence. This study aims to elucidate the impact of either depression score or depression on the risk of MCI using longitudinal data from the China Health and Retirement Longitudinal Study (CHARLS).

**Methods:**

This prospective study included 5,766 participants from CHARLS followed from 2011 to 2015. We calculated the baseline depression score using the 10-item Center for Epidemiologic Studies Depression Scale (CESD-10) and the cognitive status score after 3 years of follow-up through four dimensions: orientation, memory, calculation, and draw. We collected baseline sociological characteristics and health-related factors as covariates, using multivariate-adjusted logistics regression models (odds ratios (OR) and 95% confidence intervals (CI)) and restricted cubic splines (RCS) to estimate the effect of depressive status on MCI risk.

**Results:**

We observed 724 new cases of MCI at follow-up. Logistics regression analysis showed that participants with depression had a 58% higher risk of developing MCI than those without depression (OR = 1.58, 95%CI: 1.35-1.85), and the positive association persisted after adjusting for covariates such as sociological characteristics of the population and health-related factors (OR = 1.24, 95%CI: 1.04-1.48). We also observed a dose-response relationship between depression score and MCI risk, with participants with 11~20 and 21~30 scores having a progressively higher risk of MCI compared to participants with depression score of 0~10 (p for trend < 0.05), and a 3% increase in MCI risk for each 1-point increase in depression scores (OR = 1.03, 95%CI: 1.01-1.04). RCS analysis also showed a nonlinear association between depression score and MCI risk (p for non-linearity = 0.001), with MCI risk increasing with increasing depression score. In addition, stratified analyses based on sex, age, marital status, residence, BMI, nighttime sleep, smoking status, alcohol drinking status, baseline serological indicators, and comorbidities showed no interaction (p for interaction > 0.05) other than serum total cholesterol levels (p for interaction = 0.008).

**Conclusions:**

Among middle-aged and elderly adults from CHARLS, depression is an independent risk factor for MCI, indicating that individuals with more severe depression symptoms are more likely to suffer from MCI. Early depression screening based on CESD-10 may help identify individuals at high risk of MCI, and early intervention may reduce the incidence of MCI and Alzheimer’s disease, thereby reducing the social care burden of an ageing population.

## 1 Introduction

With the rapid development of social economy, the Chinese population is aging rapidly, according to the latest seventh population census data in 2020, China’s population aged 60 and over is 264,018,766, accounting for 18.70% of the total population, of which 190,635,280 people are aged 65 and over, accounting for 13.50% of the total population (1). Large-scale population ageing has led to a surge in the incidence, prevalence, and mortality of neurodegenerative diseases, greatly increasing the disease burden and healthcare needs of society as a whole (1). Dementia is one of the most common primary neurodegenerative diseases in the elderly, manifested by intellectual disability, personality changes, and loss of the ability to perform daily activities. Mild cognitive impairment (MCI) is a status between normal cognition and dementia that is classified as mild neurocognitive impairment by the Diagnostic and Statistical Manual of Mental Disorders, Fifth Edition (DSM-5), typically manifested by a decline in learning and memory that does not affect daily life and does not meet the diagnostic criteria for dementia (2). MCI is a heterogeneous disorder, and its incidence and prevalence are highly dependent on the diagnostic criteria applied. The prevalence of MCI in adults over 55 years of age in China is 15.4%, and the prevalence varies depending on diagnostic criteria (3). Research data show that 10%~15% of MCI patients progress to Alzheimer’s disease (AD) within 1 year, 40% within 2 years, 20%~53% within 3 years, and 55% within 4~5 years, and early detection and timely use of interventions such as aerobic exercise, mental activity, social participation, and cardiovascular risk factor control can reduce this risk (4). Therefore, identifying the risk factors for MCI can be an important means to reduce the incidence of AD (5). Studies have shown that MCI is associated with age, sex, apolipoprotein E alleles, family history, and the presence of cardiovascular risk factors such as hypertension, hyperlipidemia, coronary heart disease, and stroke (6–8).

Depression is a common neuropsychiatric disorder in older adults that primarily affects people with chronic diseases and cognitive impairment, causing distress, family breakdown, and disability, worsening the outcomes of many diseases, and increasing mortality (9). According to the latest epidemiological survey, the lifetime prevalence of depressive disorder in Chinese residents is 6.8%, of which the lifetime prevalence of depression among the elderly is 3.4% (10). According to the 2013 Global Burden of Disease study, depression has become the leading cause of the top 10 causes of disability-adjusted life years in every country worldwide (11). Studies have found that older patients with severe depression develop dementia during an episode, with some patients having resolves the symptoms of dementia after the depressive symptoms have resolved, while a large proportion of patients are left with some form of cognitive impairment after the depressive symptoms have been resolved (12), and about 40% of patients develop irreversible dementia at three years of follow-up (13). Previous studies have shown a significant positive correlation between depression and MCI (5, 14), but the lack of evidence for the Chinese population in these studies, and the vast majority of data from cross-sectional studies, making it difficult to determine the causal relationship between depression and MCI.

This study used data from the China Health and Retirement Longitudinal Study (CHARLS) to explore the prospective relationship between baseline depressive status and MCI, with a view to proposing targeted interventions for the prevention of MCI and AD.

## 2 Materials and methods

### 2.1 Study population and design

CHARLS is a nationally representative longitudinal cohort study in China. The baseline survey was conducted in 2011 to recruit respondents and their spouses from 10,257 households in 450 villages in 150 counties in 28 provinces. CHARLS interviewees conduct face-to-face personal interviews every two years. Social, demographic and lifestyle factors, as well as health-related information, were assessed using structured questionnaires. Details of CHARLS have been reported elsewhere (15). The study was approved by the Peking University Biomedical Ethics Review Committee, and all participants provided written informed consent.

**Figure 1:**
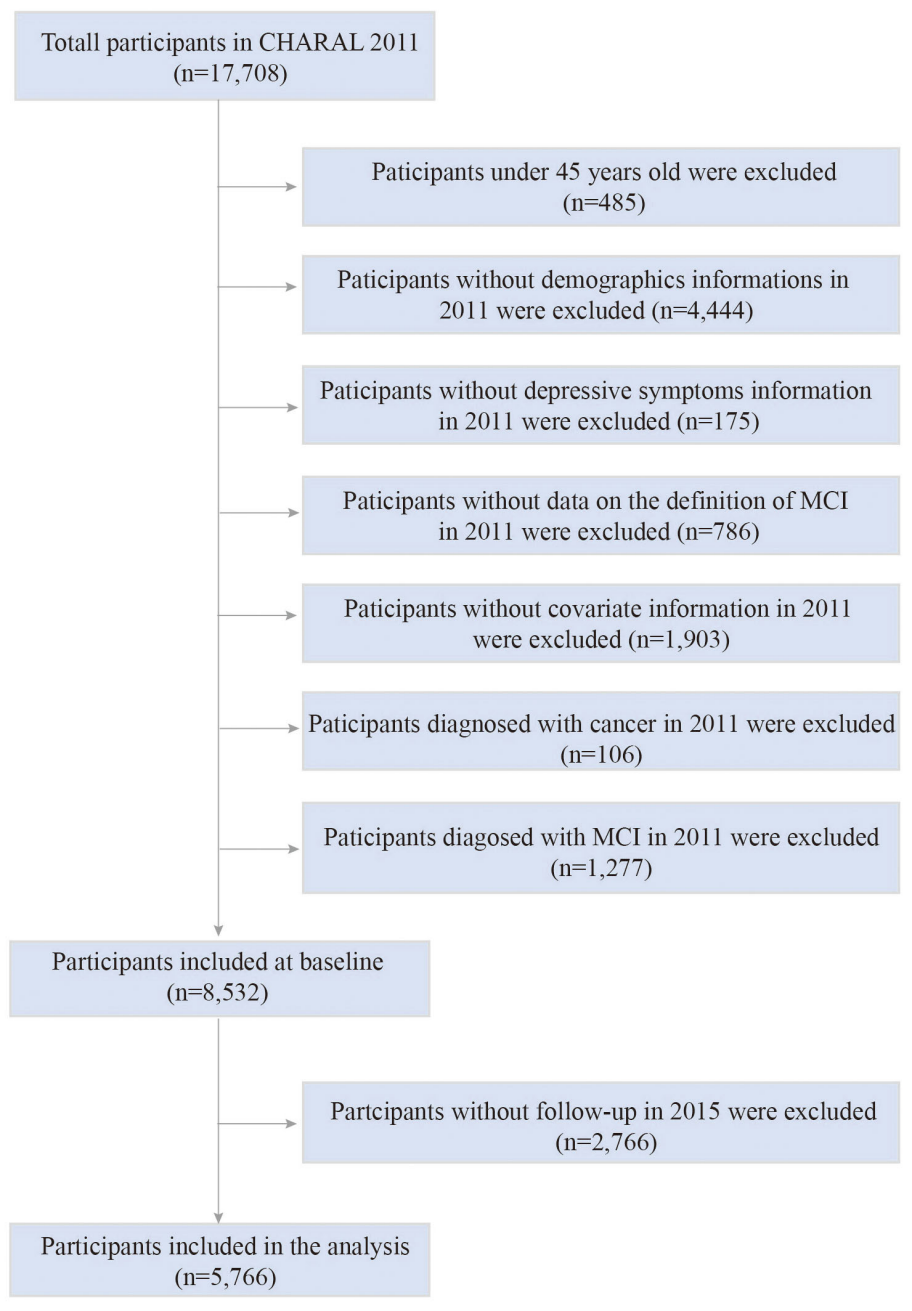
The flow chart of participants selection process. CHARLS, China Health and Retirement Longitudinal Study; MCI, mild cognitive impairment.

In this study, we retrospectively analyzed data from CHARLS in 2011 and 2015. A total of 17,708 participants were recruited in the 2011 survey. The inclusion criteria for the study were: 1) individuals who reached the age of 45 years or older in 2011; 2) have assessment data on cognitive status and depressive status. The exclusion criteria were: 1) participants who lacked relevant information about demographic data and all covariates at baseline, including gender, age, BMI, serological data, physical examination information, etc.; 2) participants with missing data from all cognitive status assessment questionnaires in 2011 and 2015; 3) participants with a diagnosis of cancer in 2011; 4) participants who met the diagnostic criteria for MCI in 2011; 5) participants with missing follow-up data in 2015. Eventually, 5,766 participants were included in the analysis. **Figure 1** shows the detailed selection process.

### 2.2 Assessment of depressive status

Depression was the main exposure indicator in this study. Depressive status is assessed by the 10-item Center for Epidemiological Research Center’s Depression Scale (CESD), which has high validity and good psychometric performance in older Chinese adults (16). Each item is assigned a score according to the degree of strength, with a minimum of 0 points and a maximum of 3 points, for a total of 10 items. The sum of the scores of all items (between 0~30) was calculated on the total depression score, and participants with a score of ≥ 10 were determined to have depression, and a score of < 10 was considered normal, and the assessment was considered invalid if there were two or more items that refused to answer (17).

### 2.3 Assessment of cognitive status

As the primary outcome, MCI was determined based on cognitive function scores referring to the modified version of Telephone Interview for Cognitive Status (TICS-m) (18). Cognitive function was assessed across two components: episodic memory and mental status (19, 20), with a combined score ranging from 0 to 31, where higher scores denote superior cognitive performance (21, 22). Episodic memory is mainly an assessment of memory dimension, conducted through face-to-face interview, with scores ranging from 0 to 20, derived from the sum of immediate (0~10 points) and delayed (0~10 points) word recall. In detail, participants were required to recall as many words as possible from a list of ten Chinese words presented by the interviewer for immediate recall and again after completing other assessments for delayed recall, with each correct word earning 1 point. Mental status, scored from 0 to 11, involved the assessment of three dimensions: orientation (0~5 points), calculation (0~5 points), and drawing (0~1 points), with orientation and calculation assessed via telephone interview and drawing assessed via face-to-face interview (21). The orientation dimension assessment items include year, month, day, day of the week, and current season, and each item is worth 1 point for correctness, with a maximum of 5 points. The computation dimension assessment asks respondents to subtract 7 from 100 5 times in a row, scoring 1 point for each 1 success, up to a maximum of 5 points. The drawing dimension assessment asks the interviewee to show the interviewee a picture of two pentagram stars overlapping each other, and asks the interviewee to draw the same pattern. If correct, 1 point is awarded. However, the diagnosis of MCI lacks a uniform standard. In this study, we employed the aging-associated cognitive decline (AACD) criteria to define MCI, which is characterized by performance at least one standard deviation (SD) below the age-specific norm (23, 24).

### 2.4 Assessment of covariates

Based on previous knowledge, we also considered the potential impact of sociodemographic characteristics and health-related factors in our studies. Sociodemographic characteristics include sex (male or female), age (continuous variable), marital status (married or otherwise), residence (urban or rural). Health-related factors include blood pressure (systolic and diastolic), body mass index (BMI, continuous variable), nighttime sleep (less than 6 hours, 6~8 hours, or more than 8 hours), smoking status (never, current, or previous), drinking status (never, current, or previous), 7 serum markers that reflect healthy status (glycosylated hemoglobin (HbA1c, continuous variable), fasting blood glucose (FBG, continuous variable), triglycerides (TG, continuous variable), total cholesterol (TC, continuous variable), high-density lipoprotein cholesterol (HDL-C, continuous variable), low-density lipoprotein cholesterol (LDL-C, continuous variable), and serum creatinine (continuous variable)), 4 cognitively related comorbidities (hypertension (yes or no), dyslipidemia (yes or no), stroke (yes or no), and diabetes/hyperglycemia (yes or no)), and common chronic disease medication use (antihypertensive drugs (yes or no), lipid-lowering drugs (yes or no), and hypoglycemic drugs (yes or no)). BMI is defined as weight (kg) divided by height (m) squared. In addition, to account for the impact of renal function, we calculated the estimated glomerular filtration rate (eGFR) using the Chinese modification of diet in renal disease (C-MDRD) formula (25).

### 2.5 Statistical analysis

For the baseline characteristics of the study population, the normal distribution quantitative data were presented as mean and standard deviation (SD), and independent sample t-test was used to compare the differences between groups; Non-normal distribution quantitative data were presented as median and interquartile range (IQR), and the Mann-Whitney U test was used to compare differences between groups; Qualitative data were presented as number and percentage, and differences between groups were compared using the Pearson chi-square test.

The prospective association of depression at baseline with new-onset MCI was analyzed using logistics regression analysis, expressed as odds ratios (OR) and 95% confidence intervals (CI). In order to avoid the interference of confounding factors, we conducted multivariable-adjusted models. Specifically, the crude model was a univariate regression model without additional adjustment for confounding factors. Model 1 adjusted for the sociological characteristics of the population and some health-related variables, including sex, age, marital status, residence, systolic and diastolic blood pressures, BMI, nighttime sleep, smoking status, and drinking status; Model 2 further adjusted for serum indicators, comorbidities, and medications based on Model 1, including HbA1c, FBG, TG, TC, HDL-C, LDL-C, eGFR, hypertension, dyslipidemia, stroke, diabetes or hyperglycemia, and use of antihypertensive drugs, Lipid-lowering drugs and hypoglycemic drugs.

To further explore the dose-response effect between depressive status and MCI, we explored the prospective association between depression symptoms and MCI risk using logistics regression models. We divided participants into 3 groups based on depression scores: 0~10 points (without obvious depression symptoms), 11~20 points (with mild depression symptoms) and 21~30 points (with moderate to severe depression symptoms), and calculated the estimated risk of MCI (OR and 95%CI) for each group by a logistics regression model. We also performed logistics regression using depression scores as continuous variables to explore changes in the risk of MCI for each 1-point increase in depression score. Here, we also control for confounders and conducted crude model, model 1, and model 2. The variable adjustments for each model are exactly the same as those of the previous model. To explore the potential nonlinear relationship between depression score and MCI risk, we plotted a restricted cubic spline (RCS) curve to describe the dynamic trend of MCI risk as depression score increased. The number of knots for RCS analysis was set to 4, at 5%, 35%, 65%, and 95%, respectively, the reference value was set to the median (OR=1.00), and the adjustment of the covariate was exactly the same as for model 2.

In addition, we conducted stratified analyses to assess potential interactions among various variables. Specifically, we examined interactions based on the following factors: sex, age, marital status, residence, BMI, nighttime sleep, smoking status, drinking status, HbA1C, FBG, TG, HDL-C, LDL-C, eGFR, and the history of disease such as hypertension, dyslipidemia, diabetes or hyperglycemia, and stroke. To evaluate these interactions, we employed likelihood ratio tests, which allowed us to determine whether the effect of baseline depression on incident MCI varied significantly across different levels of these categorical variables. For each interaction, we included an interaction term in the logistic regression model, allowing us to assess how the relationship between baseline depression and MCI at follow-up was modified by each variable. It is worth noting that we only calculated the overall p value for interaction effect in the subgroup analysis of multi-categorical variables such as nighttime sleep, smoking status, and drinking status and did not further consider the correction for multiple testing.

All statistical analyses were performed using R version 4.4.1. For hypothesis testing, a two-tailed p value of < 0.05 was considered statistically significant.

## 3 Results

### 3.1 Baseline characteristics

Our study included a total of 5,766 participants with a mean age of 58.3 years, of whom 2,104 were identified as depression at baseline. **Table 1** describes the baseline characteristics of participants divided by depression. Participants with depression tend to be older, married, and have lower levels of BMI, TG, serum creatinine, and eGFR, and higher levels of HDL-C than those without depression at baseline. They were also more likely to be female, rural residents, with diseases such as hypertension, dyslipidemia, stroke, diabetes or blood glucose abnormalities and treated with long-term antihypertensive drugs, lipid-lowering drugs, or hypoglycemic drugs, and less likely to be drinkers or abstainers, smokers or quit-smoking, and those who slept too much at night (all p < 0.05). In addition, participants suffered from depression at baseline in 2011 had generally lower cognitive scores at follow-up in 2015.

**Table 1:** Baseline characteristics of 5,766 participants according to baseline depression and new-onset MCI.

Characteristic	Overall	Depression at baseline in 2011		*P* value※	New-onset MCI at follow-up in 2015		*P* value※
	n = 5766	No (n = 3662)	Yes (n = 2104)		No (n = 5042)	Yes (n = 724)	
Sex, n (%)				<0.001			<0.001
Male	2737 (47.5)	1927 (52.6)	810 (38.5)		2477 (49.1)	260 (35.9)	
Female	3029 (52.5)	1735 (47.4)	1294 (61.5)		2565 (50.9)	464 (64.1)	
Age, mean (SD), year	58.30 (8.72)	58.02 (8.72)	58.78 (8.72)	0.002	57.44 (8.24)	64.25 (9.62)	<0.001
Marital status, n (%)				<0.001			<0.001
Married	5205 (90.3)	3362 (91.8)	1843 (87.6)		4628 (91.8)	577 (79.7)	
Other	561 (9.7)	300 (8.2)	261 (12.4)		414 (8.2)	147 (20.3)	
Residence, n (%)				<0.001			<0.001
Urban	926 (16.1)	687 (18.8)	239 (11.4)		873 (17.3)	53 (7.3)	
Rural	4840 (83.9)	2975 (81.2)	1865 (88.6)		4169 (82.7)	671 (92.7)	
BMI, mean (SD), kg/m^2^	23.79 (3.85)	24.00 (3.84)	23.41 (3.85)	<0.001	24.15 (4.05)	22.91 (4.25)	<0.001
Blood pressure, mean (SD), mmHg							
Systolic	129.14 (21.36)	129.51 (21.10)	128.49 (21.80)	0.081	128.44 (20.96)	133.98 (23.43)	<0.001
Diastolic	75.54 (12.37)	75.85 (12.20)	75.00 (12.65)	0.012	75.55 (12.37)	75.45 (12.38)	0.835
Nighttime sleep, n (%)				<0.001			<0.001
Sleepless (< 6 hours)	1644 (28.5)	731 (20.0)	913 (43.4)		1384 (27.4)	260 (35.9)	
Normal (6~8 hours)	3678 (63.8)	2630 (71.8)	1048 (49.8)		3298 (65.4)	380 (52.5)	
Oversleep (> 8 hours)	444 (7.7)	301 (8.2)	143 (6.8)		360 (7.1)	84 (11.6)	
Smoking status, n (%)				<0.001			0.034
Never	3496 (60.6)	2131 (58.2)	1365 (64.9)		3025 (60.0)	471 (65.1)	
Current	1767 (30.6)	1199 (32.7)	568 (27.0)		1570 (31.1)	197 (27.2)	
Previous	503 (8.7)	332 (9.1)	171 (8.1)		447 (8.9)	56 (7.7)	
Drinking status, n (%)				<0.001			<0.001
Never	3376 (58.6)	2081 (56.8)	1295 (61.5)		2889 (57.3)	487 (67.3)	
Current	1920 (33.3)	1338 (36.5)	582 (27.7)		1757 (34.8)	163 (22.5)	
Previous	470 (8.2)	243 (6.6)	227 (10.8)		396 (7.9)	74 (10.2)	
Blood indicators in 2011, mean (SD)							
HbA1c, %	5.26 (0.79)	5.25 (0.76)	5.28 (0.84)	0.213	5.25 (0.77)	5.30 (0.91)	0.149
FBG, mg/dl	109.82 (35.59)	109.72 (34.52)	110.01 (37.37)	0.761	109.56 (34.51)	111.68 (42.30)	0.134
TG, median [IQR], mg/dl	106.20 [75.22, 156.65]	107.08 [76.11, 158.41]	104.43 [74.34, 152.22]	0.041*	107.08 [75.22, 157.53]	102.66 [75.00, 146.02]	0.046*
TC, mg/dl	193.17 (38.23)	192.95 (38.89)	193.55 (37.06)	0.565	192.97 (37.55)	194.55 (42.66)	0.301
HDL-C, mg/dl	50.64 (15.16)	49.95 (14.92)	51.85 (15.50)	<0.001	50.46 (15.18)	51.92 (14.95)	0.015
LDL-C, mg/dl	115.97 (34.45)	115.85 (34.97)	116.16 (33.54)	0.743	115.88 (34.29)	116.58 (35.61)	0.61
Creatinine, mg/dl	0.78 (0.19)	0.79 (0.19)	0.75 (0.18)	<0.001	0.78 (0.18)	0.77 (0.21)	0.132
eGFR, ml/min/1.73 m^2^	59.66 (31.10)	62.13 (30.91)	55.35 (30.96)	<0.001	60.95 (30.41)	50.66 (34.24)	<0.001
History of comorbidities, n (%)							
Hypertension	1496 (25.9)	878 (24.0)	618 (29.4)	<0.001	1278 (25.3)	218 (30.1)	0.007
Dyslipidemia	587 (10.2)	343 (9.4)	244 (11.6)	0.008	537 (10.7)	50 (6.9)	0.002
Diabetes or Hyperglycemia	349 (6.1)	202 (5.5)	147 (7.0)	0.028	304 (6.0)	45 (6.2)	0.910
Stroke	110 (1.9)	51 (1.4)	59 (2.8)	<0.001	99 (2.0)	11 (1.5)	0.502
History of medication use, n (%)							
Hypertension drugs	1133 (19.6)	655 (17.9)	478 (22.7)	<0.001	970 (19.2)	163 (22.5)	0.043
Dyslipidemia drugs	327 (5.7)	183 (5.0)	144 (6.8)	0.004	294 (5.8)	33 (4.6)	0.194
Diabetes drugs	345 (6.0)	194 (5.3)	151 (7.2)	0.005	312 (6.2)	33 (4.6)	0.100
Depression and cognitive scores							
CES-D score in 2011, median [IQR] ^a^	7.00 [3.00, 12.00]	4.00 [2.00, 7.00]	14.00 [11.00, 18.00]	<0.001*	7.00 [3.00, 12.00]	9.00 [5.00, 14.00]	<0.001*
Cognitive score in 2015, median [IQR] ^b^	14.00 [10.00, 18.00]	15.00 [11.00, 18.00]	13.00 [9.00, 17.00]	<0.001*	15.00 [12.00, 18.00]	4.00 [2.00, 5.00]	<0.001*

※ P value calculated using Pearson χ2 test or independent samples Student^’^s t-test where appropriate. * P value calculated using Mann-Whitney U test. The eGFR calculated using modification formula of diet in renal disease. ^a^ The CES-D score was assessed at baseline to identify participants with depression. ^b^ The cognitive score was assessed at follow-up to identify new-onset MCI cases. MCI, mild cognitive impairment; BMI, body mass index; HbA1c, glycated hemoglobin; FBG, fast blood glucose; TG, triglyceride; TC, total cholesterol; HDL-C, high-density lipoprotein cholesterol; LDL-C, low-density lipoprotein cholesterol; eGFR, estimated glomerular filtration ratio; CES-D, Center for Epidemiologic Studies Depression Scale; SD, standard deviation; IQR, interquartile range.

To provide a more comprehensive baseline characteristic understanding, **Table 1** also shows the baseline characteristics according to new-onset MCI at follow-up. Participants who developed new MCI were predominantly older, married rural women. They exhibited lower BMI and eGFR, along with higher systolic blood pressure and HDL-C, and a history of hypertension at baseline. Furthermore, individuals who developed MCI had markedly higher baseline depression scores.

### 3.2 Depressive status and the risk of incident MCI

**Table 2** shows the association between depressive status and MCI risk. Based on the depression score at baseline, we divided participants into depression and non-depression groups, with a total of 334 patients with MCI identified in the depression group and 390 patients in the non-depression group at follow-up. In the crude model without covariate adjustment, participants with depression had a 58% higher risk of developing MCI than those without depression (OR = 1.58, 95%CI: 1.35-1.85). This association was slightly weakened in Model 1, which adjusted for sociological characteristics of the population and some health-related factors (OR = 1.24, 95%CI: 1.04-1.48). After further adjustment of baseline serum indicators, comorbidities, and medication use, there was no change in the estimated relative risk of MCI.

**Table 2:** Associations of depressive status with mild cognitive impairment.

Depressive status	No. of new-onset MCI / totals	Risk of MCI (OR and 95% CI)		
		Crude model	Adjusted model 1※	Adjusted model 2*
Depression				
No	390 / 3662	1.00 (Reference)	1.00 (Reference)	1.00 (Reference)
Yes	334 / 2104	1.58 (1.35-1.85)	1.24 (1.04-1.48)	1.24 (1.04-1.48)
Score of CES-D				
0~10 ^a^	426 / 3939	1.00 (Reference)	1.00 (Reference)	1.00 (Reference)
11~20 ^b^	240 / 1510	1.56 (1.31-1.85)	1.21 (1.01-1.47)	1.22 (1.01-1.47)
21~30 ^c^	58 / 317	1.85 (1.35-2.48)	1.36 (0.97-1.88)	1.37 (0.98-1.90)
*P* for trend	–	<0.001	0.014	0.013
OR (continuous)	724 / 5766	1.04 (1.03-1.06)	1.03 (1.01-1.04)	1.03 (1.01-1.04)

※ Age, sex, marital status, residence, blood pressure, BMI, nighttime sleep, smoking status, and drinking status were adjusted in model 1. * HbA1c, FBG, TG, TC, HDL_C, LDL_C, eGFR, history of hypertension, dyslipidemia, diabetes or hyperglycemia, and stroke, and medication history of hypertension, dyslipidemia, and diabetes were further adjusted in model 2. ^a^ Representing participant without obvious depression symptoms. ^b^ Representing participant with mild depression symptoms. ^c^ Representing participant with moderate to severe depression symptoms. MCI, mild cognitive impairment; OR, odds ratio; CI, confidence interval; CES-D, Center for Epidemiologic Studies Depression Scale; BMI, body mass index; HbA1c, glycated hemoglobin; FBG, fast blood glucose; TG, triglyceride; TC, total cholesterol; HDL-C, high-density lipoprotein cholesterol; LDL-C, low-density lipoprotein cholesterol; eGFR, estimated glomerular filtration ratio.

**Figure 2:**
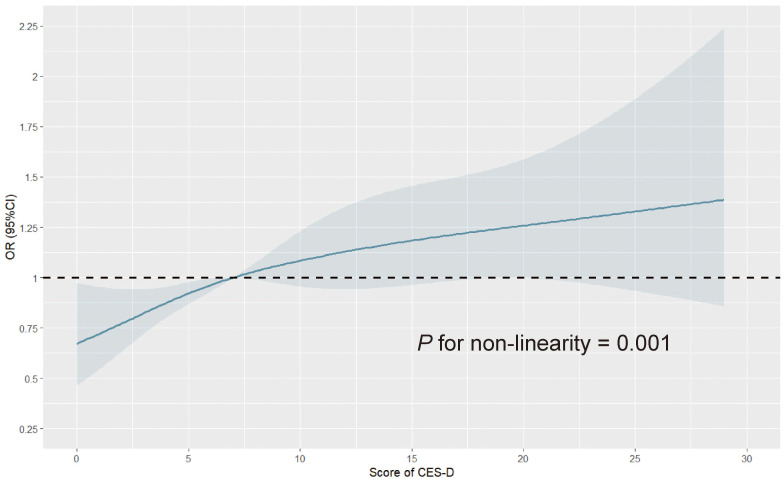
Restricted cubic spline curve for depression score and the risk of mild cognitive impairment. The number of knots for restricted spline analysis was set to 4, at 5%, 35%, 65%, and 95%, respectively, the reference value was set to the median, and covariates including sex, marital status, residence, blood pressure, BMI, nighttime sleep, smoking status, drinking status, HbA1c, FBG, TG, TC, HDL-C, LDL-C, eGFR, history of hypertension, dyslipidemia, diabetes or hyperglycemia, and stroke, and medication history of hypertension, dyslipidemia, and diabetes were adjusted. OR, odds ratio; CI, confidence interval; CES-D, Center for Epidemiologic Studies Depression Scale; BMI, body mass index; HbA1c, glycated hemoglobin; FBG, fast blood glucose; TG, triglyceride; TC, total cholesterol; HDL-C, high-density lipoprotein cholesterol; LDL-C, low-density lipoprotein cholesterol; eGFR, estimated glomerular filtration ratio.

To further explore the dose-response relationship between depression symptoms and MCI risk, we divided participants into three groups according to depression scores: 0~10 (without obvious depression symptoms), 11~20 (with mild depression symptoms), and 21~30 (with moderate to severe depression symptoms), and compared the risk of MCI in each group (**Table 2**). We found that in the crude model, participants with mild and moderate to severe depression symptoms had a 56% (OR = 1.56, 95%CI: 1.31-1.85) and 85% (OR = 1.85, 95%CI: 1.35-2.48) higher risk of MCI compared to participants without obvious depression symptoms, and there was a statistically significant difference in the increase of MCI risk as depression symptoms increased (p for trend < 0.001). After adjusting for all covariates (Model 2), the increase in MCI risk decreased, with a 22% increase for participants with mild depression symptoms and 37% for participants with moderate to severe depression symptoms, and the risk still showed a gradual increase trend (p for trend = 0.013). Moreover, we performed a regression analysis of depression score as a continuous variable and found that each 1-point increase in depression score was associated with a 3% increase in MCI risk (OR = 1.03, 95%CI: 1.01 to 1.04). In addition, we also explored a potential nonlinear relationship between depression score and MCI risk using an RCS analysis (**Figure 2**), which showed a nonlinear association (p for non-linearity = 0.001). Specifically, the risk of MCI increased with an increase in depression score, and the risk increased more rapidly before the median score (7 points) and more slowly after the median score. All the above results indicate that depression increases the risk of MCI, and the more severe depression symptoms, the higher the risk of MCI.

### 3.3 Subgroup analysis

**Figure 3:**
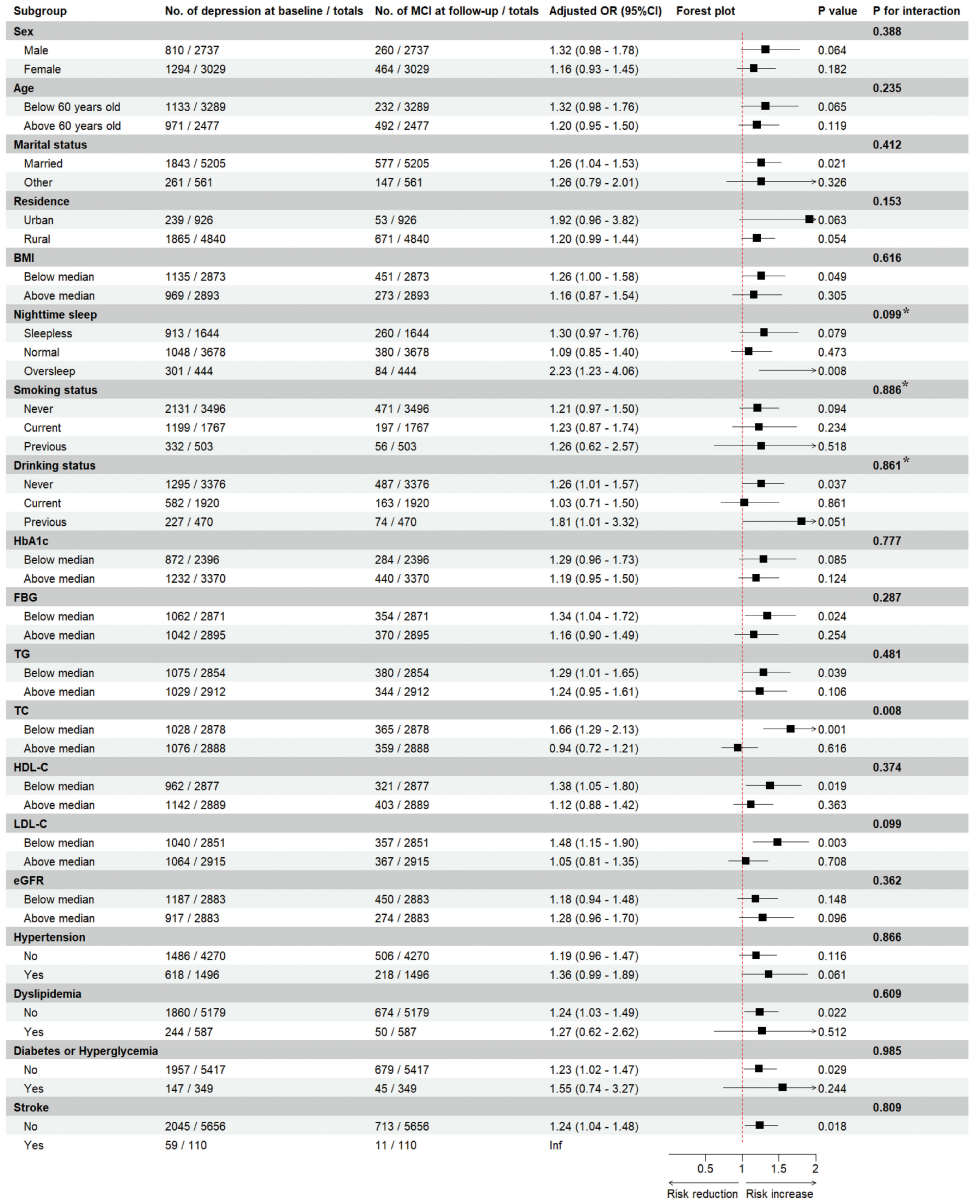
Subgroup analyses of depression and the risk of mild cognitive impairment. Estimated effects were based on the adjusted Logistics regression model adjusting for sex, marital status, residence, blood pressure, BMI, nighttime sleep, smoking status, drinking status, HbA1c, FBG, TG, TC, HDL-C, LDL-C, eGFR, history of hypertension, dyslipidemia, diabetes or hyperglycemia, and stroke, and medication history of hypertension, dyslipidemia, and diabetes were adjusted. The ORs and 95%CIs for mild cognitive impairment in the depression group were compared with the non-depression group (as a reference). For each interaction, we included an interaction term in the logistic regression model, and the P value for interaction was calculated using likelihood ratio test, with a value less than 0.05 considered to be an interaction effect. * The overall p value for a multi-categorical variable reflects the overall interaction effect of the variable, and a value ≥ 0.05 indicates that there is insufficient evidence for a significant interaction. MCI, mild cognitive impairment; OR, odds ratio; CI, confidence interval; BMI, body mass index; HbA1c, glycated hemoglobin; FBG, fast blood glucose; TG, triglyceride; TC, total cholesterol; HDL-C, high-density lipoprotein cholesterol; LDL-C, low-density lipoprotein cholesterol; eGFR, estimated glomerular filtration ratio.

We explored the interaction effect of certain confounders in the association of depression and MCI risk through subgroup analyses (**Figure 3**). We found that MCI risk did not differ by gender, age, marital status, place of residence, BMI, nighttime sleep duration, smoking status, alcohol drinking status, and serum levels of HbA1c, FBG, TG, HDL-C, LDL-C, and eGFR, as well as in comorbidities (i.e., hypertension, dyslipidemia, diabetes/hyperglycemia, and stroke) (all p for interaction > 0.05), but by high or low serum TC levels (p for interaction = 0.008). Specifically, depression was associated with a 66% increased risk of MCI in participants with lower serum TC levels (OR = 1.66, 95%CI: 1.29-2.13), but not significantly in participants with higher TC levels (OR = 0.94, 95%CI: 0.72-1.21).

## 4 Discussion

To our knowledge, this study is the first prospective study to use nationally representative data to examine the association of depressive status with MCI risk in middle-aged and elderly Chinese adults from CHARLS study. We analyzed potential linear and nonlinear associations between the two, and found that baseline depression was independent risk factor for new-onset MCI, and individuals with more severe depression symptoms were more likely to suffer from MCI. This finding implies that early depression screening based on 10-item CESD and timely intervention of positive individuals may be an effective way to prevent MCI, and focused screening of MCI symptoms in individuals with depression is an effective way to achieve early diagnosis and treatment of MCI.

The overall cognitive function assessment encompasses multiple cognitive domains, providing a comprehensive understanding of the examinee’s cognitive status and characteristics. The Mini-Mental State Examination (MMSE) and the Montreal Cognitive Assessment (MoCA) are commonly used tools for cognitive evaluation in clinical settings. The MMSE is one of the most widely utilized cognitive assessment scales, covering domains such as orientation, memory, attention, calculation, language use, and visuospatial skills. A meta-analysis indicated that the sensitivity of the MMSE for screening cognitive impairment in primary care settings was 0.64, with a specificity of 0.80. The area under the receiver operating characteristic curve (AUC) for distinguishing normal cognition from MCI in the elderly ranged from 0.43 to 0.94, while the AUC for detecting AD ranged from 0.67 to 0.99 (26), suggesting that the MMSE is suitable for large-scale screening of dementia patients, though it has limitations in differentiating between normal cognition and MCI, as well as between MCI and dementia. In contrast, the MoCA encompasses a broader range of cognitive domains, including memory, language, attention, abstract thinking, orientation, visuospatial skills, and executive function. A meta-analysis showed that the AUC for the MoCA in distinguishing normal cognition from MCI was between 0.71 and 0.99, and between 0.87 and 0.99 for detecting AD (26), indicating that the MoCA is more sensitive than the MMSE for screening MCI and effectively detecting cognitive heterogeneity (27). In this study, we assessed cognitive function using TICS-m method, employing the AACD criteria for diagnosing MCI. The original TICS based on the MMSE included 11 items covering orientation, memory, calculation, attention, language, repetition, general knowledge, and abstract thinking, with a maximum score of 40 (28). Gallo and Breitner later revised the initial version, expanding it to TICS-m with 21 items and a total score of 50, which includes additional tasks such as word delayed recall and tapping a telephone five times (29). A large-scale telephone screening of 12,709 elderly twins using TICS-m demonstrated a sensitivity of 99% and a specificity of 86%, making it more suitable for early detection of cognitive impairment compared to the original TICS. Studies comparing the screening capabilities of TICS-m, MMSE, and MoCA for MCI and AD have shown that TICS and TICS-m possess comparable reliability and validity to both MMSE and MoCA in screening elderly individuals for cognitive impairment (30–32). Consequently, this study opted for the TICS-m method, incorporating the delayed word recall component. Notably, due to the limitations of the original CHARLS questionnaire, our assessment did not fully align with TICS-m but focused on evaluating memory, orientation, calculation, and drawing, resulting in a cognitive score ranging from 0 to 31 to represent overall cognitive function. Although this may affect the accuracy of MCI diagnosis, its effectiveness and feasibility have been well established in multiple studies (21, 22).

Research evidence on depression and MCI is limited, and previous studies have focused on associations between depression and cognitive impairment or dementia. Depression and cognitive impairment often coexist in older adults, and the relationship between the two is extremely complex (33). Cognitive impairment is a core feature of depression (34), while depression, anxiety, and apathy are common in MCI and are potential risk factors for cognitive decline and dementia (35). González Hernández et al. (36) explored in depth the question of whether depression is a priority over the emergence of MCI as a risk factor for AD through a comprehensive literature review. They found that depression was a risk factor for AD, but not a predictor of AD disease progression, and that its association with AD was significantly enhanced when depressive symptoms were combined with MCI. Our prospective study provides further evidence that depression and MCI may not occur at the same time, and that pre-existing depression increases the risk of subsequent MCI, which in turn develops into a comorbid status of depression and MCI.

Notably, the results of the subgroup analysis showed that the positive association between depression and MCI risk was significant in participants with lower serum TC levels, but not in the higher participants. The relationship between serum TC level and the risks of depression and cognitive impairment is currently highly controversial. There is evidence linking high TC levels to high morbidity and poor prognosis of depression and cognitive impairment (37–39), although the conclusions of these studies are mostly based on cross-sectional data. However, there have also been studies that have reported a positive association between low serum cholesterol level and the risks of depression and cognitive impairment. The results of a large longitudinal study by Partonen et al. (40) showed that low serum TC level was associated with depressed mood and could be used to predict severe outcomes such as major depression and suicide. In addition, Park et al. (41) found that persistently low or elevated total cholesterol levels during adolescence may increase the risk of depressive symptoms in early adulthood, while Cepeda et al. (42) and Zhang et al. (43) showed that low cholesterol levels and cholesterol types were not associated with depression. Similarly, one study from South Korea (44) showed that high TC level was not associated with an increased risk of depression, but significantly associated with cognitive impairment. Therefore, the effect of serum TC level on the risks of depression and cognitive impairment deserves further validation in high-quality prospective and experimental studies.

There are some strengths to this study. First, the study data were drawn from a nationally representative general population with large sample size, providing prospective evidence for a hypothetical association between depressive status and MCI. Second, we comprehensively explored the effect of depressive status on MCI risk, with the logistics regression model showing a linear positive association, and the RCS curve showing a nonlinear association. Third, we tried to control for potential confounding factors such as sociological characteristics and health-related factors as much as possible in the data analysis process, and we conducted a series of subgroup analyses to improve the reliability of the results. Finally, and most importantly, our research has important practical implications. We provide prospective evidence that depression increases the risk of MCI, which means that individuals with depression should be the priority population for MCI screening to improve the effectiveness of screening and achieve early diagnosis and management. Meanwhile, depression screening for middle-aged and elderly individuals is essential, as early intervention can significantly alleviate depressive symptoms, thereby reducing the occurrence of MCI. There is sufficient evidence to support that interventions such as drug treatment (45, 46), changes of lifestyle (47–51) (e.g., healthy dietary patterns, alcohol restriction, smoking cessation, physical activity, weight control, nutrient supplementation, etc.), management of chronic diseases (4, 51–54) (e.g., diabetes, hypertension, dyslipidemia, etc.), and improvement of intestinal flora (55–57) may improve cognitive dysfunction and reduce the transition from MCI to AD.

However, there are serval limitations that cannot be ignored. First, due to the data availability of CHARLS, all our analyses relied on data from only two time points (2011 and 2015), which obviously limited the ability to observe the dynamic changes in depressive status and their relationship with MCI over time. In other words, this study cannot fully confirm the potential causal relationship between baseline depressive status and new-onset MCI. Second, although we adjusted for many potential confounding factors for MCI according to previous research, interference with residual confounders, such as diet, physical activity, etc., could not be ruled out. Third, assessment of depressive status in this study were based on the self-reported 10-item CESD questionnaire, which inevitably had recall bias. Fourth, this study is aimed at the middle-aged and elderly population in China, and the generalization of the results in other regions may be limited, and future studies need to validate our conclusions in a more diverse population. Meanwhile, study population had potential selection bias, only 32.56% (5,766/17,708) of the total CHARLS population were included in the final analysis, and the prevalence of baseline depression in this study was notably higher than that in the general population. While the data originates from a nationally representative cohort, the exclusion criteria and resultant sample characteristics limited the generalizability of the findings. Fifth, the CHARLS follow-up data used in this study were collected centrally in 2015, and MCI was diagnosed centrally through the follow-up data. Due to the lack of a specific time to onset of MCI, we used a logistics regression model rather than a COX regression model. Sixth, we assessed cognitive function using the TICS-m method, which was standardized for people aged 60~98 years, which may lead to a lower reliability of MCI assessment. Finally, although the evidence for association in prospective studies is stronger than that in cross-sectional studies, there is a lack of clear biological mechanisms, so we are still unable to establish a causal association between depressive status and MCI, and further experimental studies are needed to confirm this.

## 5 Conclusion

In conclusion, we found that depression was an independent risk factor for MCI in the middle-aged and elderly Chinese adults from CHARLS study, and as depressive symptoms worsen, the risk of MCI gradually increased, providing an evidence basis for the primary prevention of MCI. Early depression screening based on 10-item CESD in community nursing services may help identify individuals at high risk of MCI, and further positive psychological and lifestyle interventions for middle-aged and elderly individuals with depression may reduce the incidence of MCI. Meanwhile, focused screening of MCI symptoms in individuals with depression may help increase the detection rate of MCI, promote early diagnosis and treatment, and reduce the transition from MCI to AD. Of course, our findings need to be further validated in more large prospective cohorts, randomized controlled trials, and experimental studies.

## Data Availability

The data that support the findings of this study are openly available in China Health and Retirement Longitudinal Study at http://charls.pku.edu.cn/index.html.

## References

[1] Ren R Qi J Lin S Liu X Yin P Wang Z. The China Alzheimer report 2022. Gen Psychiatry. (2022) 35:e100751. doi: 10.1136/gpsych-2022-100751 PMC8919463 35372787

[2] First MB. Diagnostic and statistical manual of mental disorders, 5th edition, and clinical utility. J nervous Ment Dis. (2013) 201:727–9. doi: 10.1097/NMD.0b013e3182a2168a 23995026

[3] Deng Y Zhao S Cheng G Yang J Li B Xu K. The prevalence of mild cognitive impairment among Chinese people: A meta-analysis. Neuroepidemiology. (2021) 55:79–91. doi: 10.1159/000512597 33756479

[4] Langa KM Levine DA. The diagnosis and management of mild cognitive impairment: a clinical review. JAMA. (2014) 312:2551–61. doi: 10.1001/jama.2014.13806 PMC4269302 25514304

[5] Hu M Shu X Wu X Chen F Hu H Zhang J. Neuropsychiatric symptoms as prognostic makers for the elderly with mild cognitive impairment: a meta-analysis. J Affect Disord. (2020) 271:185–92. doi: 10.1016/j.jad.2020.03.061 32479315

[6] Au B Dale-Mcgrath S Tierney MC. Sex differences in the prevalence and incidence of mild cognitive impairment: A meta-analysis. Ageing Res Rev. (2017) 35:176–99. doi: 10.1016/j.arr.2016.09.005 27771474

[7] Caselli RJ Dueck AC Osborne D Sabbagh MN Connor DJ Ahern GL. Longitudinal modeling of age-related memory decline and the APOE epsilon4 effect. New Engl J Med. (2009) 361:255–63. doi: 10.1056/NEJMoa0809437 PMC2928998 19605830

[8] Pal K Mukadam N Petersen I Cooper C. Mild cognitive impairment and progression to dementia in people with diabetes, prediabetes and metabolic syndrome: a systematic review and meta-analysis. Soc Psychiatry Psychiatr Epidemiol. (2018) 53:1149–60. doi: 10.1007/s00127-018-1581-3 PMC6208946 30182156

[9] Alexopoulos GS. Depression in the elderly. Lancet (London England). (2005) 365:1961–70. doi: 10.1016/S0140-6736(05)66665-2 15936426

[10] Geriatric Psychiatry Group C M A P B. Expert consensus on the diagnosis and treatment of depressive disorders in the elderly. Chin J Psychiatry. (2017) 50:329–34.

[11] Chinese Medical Association Chinese Medical Association Journal Chinese Medical Association General Practice Branch Chinese Medical Association Psychiatry Branch Depression Disorders Collaboration Group Chinese Medical Association "Chinese General Practitioner Journal" Editorial Committee Neurological Disease Primary Care Guidelines Writing Expert Group. Guideline for primary care of major depressive disorder: practice version (2021). Chin J Gen Practitioners. (2021) 20:1261–8. doi: 10.3760/cma.j.cn114798-20211020-00779

[12] DeFrancesco M Marksteiner J Deisenhammer EA Hinterhuber H Weiss EM. Association of mild cognitive impairment (MCI) and depression. Neuropsychiatrie: Klinik Diagnostik Therapie und Rehabilitation: Organ der Gesellschaft Osterreichischer Nervenarzte und Psychiater. (2009) 23:144–50. 19703379

[13] Alexopoulos GS Meyers BS Young RC Mattis S Kakuma T. The course of geriatric depression with “reversible dementia”: a controlled study. Am J Psychiatry. (1993) 150:1693–9. doi: 10.1176/ajp.150.11.1693 8105707

[14] Divers R Robinson A Miller L Davis K Reed C Calamia M. Examining heterogeneity in depression symptoms and associations with cognition and everyday function in MCI. J Clin Exp Neuropsychol. (2022) 44:185–94. doi: 10.1080/13803395.2022.2102154 PMC9665159 35862574

[15] Zhao Y Hu Y Smith JP Strauss J Yang G. Cohort profile: the China health and retirement longitudinal study (CHARLS). Int J Epidemiol. (2014) 43:61–8. doi: 10.1093/ije/dys203 PMC3937970 23243115

[16] Andresen EM Malmgren JA Carter WB Patrick DL. Screening for depression in well older adults: evaluation of a short form of the CES-D (Center for Epidemiologic Studies Depression Scale). Am J Prev Med. (1994) 10:77–84. doi: 10.1016/S0749-3797(18)30622-6 8037935

[17] Liu Y Cui J Cao L Stubbendorff A Zhang S. Association of depression with incident sarcopenia and modified effect from healthy lifestyle: The first longitudinal evidence from the CHARLS. J Affect Disord. (2024) 344:373–9. doi: 10.1016/j.jad.2023.10.012 37805156

[18] Welsh K Breitner J Magruder-Habib K. Detection of dementia in the elderly using telephone screening of cognitive status. Neuropsychiatry Neuropsychol Behav Neurol. (1993) 6:103–10.

[19] Luo Y Pan X Zhang Z. Productive activities and cognitive decline among older adults in China: Evidence from the China Health and Retirement Longitudinal Study. Soc Sci Med (1982). (2019) 229:96–105. doi: 10.1016/j.socscimed.2018.09.052 30274688

[20] Lei X Smith JP Sun X Zhao Y. Gender differences in cognition in China and reasons for change over time: evidence from CHARLS. J economics Ageing. (2014) 4:46–55. doi: 10.1016/j.jeoa.2013.11.001 PMC4269268 25530942

[21] Luo H Hu H Zheng Z Sun C Yu K. The impact of living environmental factors on cognitive function and mild cognitive impairment: evidence from the Chinese elderly population. BMC Public Health. (2024) 24:2814. doi: 10.1186/s12889-024-20197-2 39402570 PMC11472552

[22] Hu Y Peng W Ren R Wang Y Wang G. Sarcopenia and mild cognitive impairment among elderly adults: The first longitudinal evidence from CHARLS. J cachexia sarcopenia Muscle. (2022) 13:2944–52. doi: 10.1002/jcsm.13081 PMC9745544 36058563

[23] Levy R. Aging-associated cognitive decline. Working Party of the International Psychogeriatric Association in collaboration with the World Health Organization. Int psychogeriatrics. (1994) 6:63–8. doi: 10.1017/S1041610294001626 8054494

[24] Richards M Touchon J Ledesert B Richie K. Cognitive decline in ageing: are AAMI and AACD distinct entities? Int J geriatric Psychiatry. (1999) 14:534–40. doi: 10.1002/(sici)1099-1166(199907)14:7<534::aid-gps963>3.0.co;2-b 10440973

[25] Ma YC Zuo L Chen JH Luo Q Yu XQ Li Y. Modified glomerular filtration rate estimating equation for Chinese patients with chronic kidney disease. J Am Soc Nephrology: JASN. (2006) 17:2937–44. doi: 10.1681/ASN.2006040368 16988059

[26] Pinto TCC MaChado L Bulgacov TM Rodrigues-Júnior AL Costa MLG Ximenes RCC. Is the Montreal Cognitive Assessment (MoCA) screening superior to the Mini-Mental State Examination (MMSE) in the detection of mild cognitive impairment (MCI) and Alzheimer’s Disease (AD) in the elderly? Int psychogeriatrics. (2019) 31:491–504. doi: 10.1017/S1041610218001370 30426911

[27] Jia X Wang Z Huang F Su C Du W Jiang H. A comparison of the Mini-Mental State Examination (MMSE) with the Montreal Cognitive Assessment (MoCA) for mild cognitive impairment screening in Chinese middle-aged and older population: a cross-sectional study. BMC Psychiatry. (2021) 21:485. doi: 10.1186/s12888-021-03495-6 34607584 PMC8489046

[28] Ferrucci I del Lungo I Guralnik JM Bandinelli S Benvenuti E Salani B. Is the telephone interview for cognitive status a valid alternative in persons who cannot be evaluated by the Mini Mental State Examination? Aging Clin Exp Res. (1998) 10:332–8. doi: 10.1007/BF0333979610:332-8 9825025

[29] Gallo JJ Breitner JC. Alzheimer’s disease in the NAS-NRC Registry of aging twin veterans, IV. Performance characteristics of a two-stage telephone screening procedure for Alzheimer’s dementia. psychol Med. (1995) 25:1211–9. doi: 10.1017/s0033291700033183 8637951

[30] Seo EH Lee DY Kim SG Kim KW Kim DH Kim BJ. Validity of the telephone interview for cognitive status (TICS) and modified TICS (TICSm) for mild cognitive imparment (MCI) and dementia screening. Arch gerontology geriatrics. (2011) 52:e26–30. doi: 10.1016/j.archger.2010.04.008 20471701

[31] Chappelle SD Gigliotti C Léger GC Peavy GM Jacobs DM Banks SJ. Comparison of the telephone-Montreal Cognitive Assessment (T-MoCA) and Telephone Interview for Cognitive Status (TICS) as screening tests for early Alzheimer’s disease. Alzheimer’s dementia. (2023) 19:4599–608. doi: 10.1002/alz.v19.10 PMC10509307 36939111

[32] Zietemann V Kopczak A Müller C Wollenweber FA Dichgans M. Validation of the telephone interview of cognitive status and telephone montreal cognitive assessment against detailed cognitive testing and clinical diagnosis of mild cognitive impairment after stroke. Stroke. (2017) 48:2952–7. doi: 10.1161/STROKEAHA.117.017519 29042492

[33] Mukku SSR Dahale AB Muniswamy NR Muliyala KP Sivakumar PT Varghese M. Geriatric depression and cognitive impairment-an update. Indian J psychol Med. (2021) 43:286–93. doi: 10.1177/0253717620981556 PMC8327864 34385720

[34] Rock PL Roiser JP Riedel WJ Blackwell AD. Cognitive impairment in depression: a systematic review and meta-analysis. psychol Med. (2014) 44:2029–40. doi: 10.1017/S0033291713002535 24168753

[35] Ma L. Depression, anxiety, and apathy in mild cognitive impairment: current perspectives. Front Aging Neurosci. (2020) 12:9. doi: 10.3389/fnagi.2020.00009 32082139 PMC7002324

[36] González Hernández A Rodríguez Quintero AM Bonilla Santos J. Depression and its relationship with mild cognitive impairment and Alzheimer disease: A review study. Rev espanola geriatria y gerontologia. (2022) 57:118–28. doi: 10.1016/j.regg.2021.10.002 34848100

[37] Ozturk HM Ogan N Erdogan M Akpinar EE Ilgar C Ozturk S. The association between total cholesterol and cognitive impairment in chronic obstructive pulmonary disease patients. Prostaglandins other Lipid Mediators. (2023) 164:106697. doi: 10.1016/j.prostaglandins.2022.106697 36347442

[38] Wang Y Shen R. Association of remnant cholesterol with depression among US adults. BMC Psychiatry. (2023) 23:259. doi: 10.1186/s12888-023-04770-4 37069633 PMC10108798

[39] Vilibić M Jukić V Pandžić-Sakoman M Bilić P Milošević M Association between total serum cholesterol and depression, aggression, and suicidal ideations in war veterans with posttraumatic stress disorder: a cross-sectional study. Croatian Med J. (2014) 55:520–9. doi: 10.3325/cmj.2014.55.520 PMC4228297 25358885

[40] Partonen T Haukka J Virtamo J Taylor PR Lönnqvist J. Association of low serum total cholesterol with major depression and suicide. Br J Psychiatry. (1999) 175:259–62. doi: 10.1192/bjp.175.3.259 10645328

[41] Park JH Jung SJ Jung Y Ahn SV Lee E Kim HC. Association between the change of total cholesterol during adolescence and depressive symptoms in early adulthood. Eur Child Adolesc Psychiatry. (2021) 30:261–9. doi: 10.1007/s00787-020-01511-w 32193646

[42] Cepeda MS Kern DM Blacketer C Drevets WC. Low levels of cholesterol and the cholesterol type are not associated with depression: Results of a cross-sectional NHANES study. J Clin lipidology. (2020) 14:515–21. doi: 10.1016/j.jacl.2020.06.001 32622845

[43] Zhang Q Liu Z Wang Q Li X. Low cholesterol is not associated with depression: data from the 2005-2018 National Health and Nutrition Examination Survey. Lipids Health Dis. (2022) 21:35. doi: 10.1186/s12944-022-01645-7 35369876 PMC8978383

[44] Han KT Kim SJ. Are serum cholesterol levels associated with cognitive impairment and depression in elderly individuals without dementia?: A retrospective cohort study in South Korea. Int J geriatric Psychiatry. (2021) 36:163–73. doi: 10.1002/gps.v36.1 32830355

[45] Blackman J Swirski M Clynes J Harding S Leng Y Coulthard E. Pharmacological and non-pharmacological interventions to enhance sleep in mild cognitive impairment and mild Alzheimer’s disease: A systematic review. J sleep Res. (2021) 30:e13229. doi: 10.1111/jsr.13229 33289311 PMC8365694

[46] Craft S Raman R Chow TW Rafii MS Sun CK Rissman RA. Safety, efficacy, and feasibility of intranasal insulin for the treatment of mild cognitive impairment and Alzheimer disease dementia: A randomized clinical trial. JAMA Neurol. (2020) 77:1099–109. doi: 10.1001/jamaneurol.2020.1840 PMC7309571 32568367

[47] Ornish D Madison C Kivipelto M Kemp C McCulloch CE Galasko D. Effects of intensive lifestyle changes on the progression of mild cognitive impairment or early dementia due to Alzheimer’s disease: a randomized, controlled clinical trial. Alzheimer’s Res Ther. (2024) 16:122. doi: 10.1186/s13195-024-01482-z 38849944 PMC11157928

[48] Kivipelto M Mangialasche F Ngandu T. Lifestyle interventions to prevent cognitive impairment, dementia and Alzheimer disease. Nat Rev Neurol. (2018) 14:653–66. doi: 10.1038/s41582-018-0070-3 30291317

[49] Dominguez LJ Veronese N Vernuccio L Catanese G Inzerillo F Salemi G. Nutrition, physical activity, and other lifestyle factors in the prevention of cognitive decline and dementia. Nutrients. (2021) 13(11):4080. doi: 10.3390/nu13114080 34836334 PMC8624903

[50] Duan H Zhou D Xu N Yang T Wu Q Wang Z. Association of unhealthy lifestyle and genetic risk factors with mild cognitive impairment in Chinese older adults. JAMA network Open. (2023) 6:e2324031. doi: 10.1001/jamanetworkopen.2023.24031 37462970 PMC10354670

[51] Song D Yu DSF. Effects of a moderate-intensity aerobic exercise programme on the cognitive function and quality of life of community-dwelling elderly people with mild cognitive impairment: A randomised controlled trial. Int J Nurs Stud. (2019) 93:97–105. doi: 10.1016/j.ijnurstu.2019.02.019 30901716

[52] Santisteban MM Iadecola C Carnevale D. Hypertension, neurovascular dysfunction, and cognitive impairment. Hypertension (Dallas Tex: 1979). (2023) 80:22–34. doi: 10.1161/HYPERTENSIONAHA.122.18085 36129176 PMC9742151

[53] Ungvari Z Toth P Tarantini S Prodan CI Sorond F Merkely B. Hypertension-induced cognitive impairment: from pathophysiology to public health. Nat Rev Nephrol. (2021) 17:639–54. doi: 10.1038/s41581-021-00430-6 PMC8202227 34127835

[54] Zilliox LA Chadrasekaran K Kwan JY Russell JW. Diabetes and cognitive impairment. Curr Diabetes Rep. (2016) 16:87. doi: 10.1007/s11892-016-0775-x PMC5528145 27491830

[55] Fan X Zhang Y Song Y Zhao Y Xu Y Guo F. Compound Danshen Dripping Pills moderate intestinal flora and the TLR4/MyD88/NF-κB signaling pathway in alleviating cognitive dysfunction in type 2 diabetic KK-Ay mice. Phytomedicine. (2023) 111:154656. doi: 10.1016/j.phymed.2023.154656 36682300

[56] Zhang Q Zhao W Hou Y Song X Yu H Tan J. β-Glucan attenuates cognitive impairment of APP/PS1 mice via regulating intestinal flora and its metabolites. CNS Neurosci Ther. (2023) 29:1690–704. doi: 10.1111/cns.14132 PMC10173722 36890624

[57] Den H Dong X Chen M Zou Z. Efficacy of probiotics on cognition, and biomarkers of inflammation and oxidative stress in adults with Alzheimer’s disease or mild cognitive impairment - a meta-analysis of randomized controlled trials. Aging. (2020) 12:4010–39. doi: 10.18632/aging.102810 PMC7066922 32062613

